# Heat Stress: Effects on Rumen Microbes and Host Physiology, and Strategies to Alleviate the Negative Impacts on Lactating Dairy Cows

**DOI:** 10.3389/fmicb.2022.804562

**Published:** 2022-02-28

**Authors:** Seon Ho Kim, Sonny C. Ramos, Raniel A. Valencia, Yong Il Cho, Sang Suk Lee

**Affiliations:** ^1^Ruminant Nutrition and Anaerobe Laboratory, Department of Animal Science and Technology, Sunchon National University, Suncheon, South Korea; ^2^Department of Animal Science, College of Agriculture, Central Luzon State University, Science City of Muñoz, Philippines; ^3^Animal Disease and Diagnostic Laboratory, Department of Animal Science and Technology, Sunchon National University, Suncheon, South Korea

**Keywords:** heat stress, dairy cows, gut microbiome, physiology, metabolism, alleviation strategies

## Abstract

Heat stress (HS) in dairy cows causes considerable losses in the dairy industry worldwide due to reduced animal performance, increased cases of metabolic disorders, altered rumen microbiome, and other health problems. Cows subjected to HS showed decreased ruminal pH and acetate concentration and an increased concentration of ruminal lactate. Heat-stressed cows have an increased abundance of lactate-producing bacteria such as *Streptococcus* and unclassified Enterobacteriaceae, and soluble carbohydrate utilizers such as *Ruminobacter*, *Treponema*, and unclassified Bacteroidaceae. Cellulolytic bacteria, especially Fibrobacteres, increase during HS due to a high heat resistance. Actinobacteria and *Acetobacter*, both acetate-producing bacteria, decreased under HS conditions. Rumen fermentation functions, blood parameters, and metabolites are also affected by the physiological responses of the animal during HS. Isoleucine, methionine, myo-inositol, lactate, tryptophan, tyrosine, 1,5-anhydro-D-sorbitol, 3-phenylpropionic acid, urea, and valine decreased under these conditions. These responses affect feed consumption and production efficiency in milk yield, growth rate, and reproduction. At the cellular level, activation of heat shock transcription factor (HSF) (located throughout the nucleus and the cytoplasm) and increased expression of heat shock proteins (HSPs) are the usual responses to cope with homeostasis. HSP70 is the most abundant HSP family responsible for the environmental stress response, while HSF1 is essential for increasing cell temperature. The expression of bovine lymphocyte antigen and histocompatibility complex class II (DRB3) is downregulated during HS, while HSP90 beta I and HSP70 1A are upregulated. HS increases the expression of the cytosolic arginine sensor for mTORC1 subunits 1 and 2, phosphorylation of mammalian target of rapamycin and decreases the phosphorylation of Janus kinase-2 (a signal transducer and activator of transcription factor-5). These changes in physiology, metabolism, and microbiomes in heat-stressed dairy cows require urgent alleviation strategies. Establishing control measures to combat HS can be facilitated by elucidating mechanisms, including proper HS assessment, access to cooling facilities, special feeding and care, efficient water systems, and supplementation with vitamins, minerals, plant extracts, and probiotics. Understanding the relationship between HS and the rumen microbiome could contribute to the development of manipulation strategies to alleviate the influence of HS. This review comprehensively elaborates on the impact of HS in dairy cows and introduces different alleviation strategies to minimize HS.

## Introduction

Recent research has confirmed that microbiome is fundamental to health function and affects almost every aspect of an animal’s physiology. In dairy cows, the rumen contains highly abundant and diverse microbes that play a significant role in the host’s metabolism and health (Kim et al., 2020). The structure and functioning of the rumen microbiome are affected by physical and chemical factors, such as diet (McCann et al., 2014), feeding programs (Golder et al., 2014), environment (Jami et al., 2013), feeding behavior (Prendiville et al., 2010), and individual characteristics (Weimer et al., 2010). Thus, environmental factors such as increasing temperatures may affect animal welfare through direct biological effects and through the disruption of mutualistic relationships between the animals and microbiomes (Buse et al., 1999; Sepulveda and Moeller, 2020). During heat stress (HS), animals show various physiological, endocrine, and behavioral mechanisms to cope with HS. Normally, decreased feed intake due to HS is considered a main factor resulting in a negative energy balance and decreased milk production (Sammad et al., 2020).

Dairy cows are among the most sensitive domestic animals to HS, which creates a global challenge for the dairy industry (Tian et al., 2015). According to Zhao et al. (2019), the health and milk production of dairy cows is negatively affected by HS, and the gut microbiome plays an important role in these effects. During HS, dairy cows are at risk of rumen acidosis (Sammad et al., 2020) as ruminal pH decreases due to high concentration and accumulation of lactic acid (Yadav et al., 2013). Low pH conditions decrease the abundance of fibrolytic bacteria, causing fiber digestibility reduction (Baek et al., 2020). High feed concentrate intake rather than forage (Uyeno et al., 2010) decreases rumen motility and rumination, which lowers saliva production as a buffering agent in the rumen (Das et al., 2016), thus lowering rumen pH. Consequently, an increase in lactate and decrease in acetate-producing species in the gut microbial community is a potential reason for the decline in milk production (Zhao et al., 2019). Meanwhile, only a few studies have assessed the impact of HS on the rumen microbiome (Zhao et al., 2019; Kim et al., 2020; Patra and Kar, 2021).

While considering the usual physiological and metabolic changes of heat-stressed animals, it is also important to understand the relationship between HS and the gut microbiome to help develop strategies to alleviate the effect of HS by manipulating the ruminal microbial composition. These physiological and metabolic changes include altered immune functions; increased expression of heat shock proteins (HSPs); increase in body temperature, respiration, non-esterified fatty acid (NEFA), blood urea nitrogen (BUN), and ketone bodies; and decreased feed intake, blood glucose, cholesterol, and mineral blood concentrations (Sammad et al., 2020; Yue et al., 2020). Thus, this review comprehensively elaborates the impact of HS on the gut microbiome, its effect on the physiology and metabolism of dairy cows and introduces different alleviation strategies to minimize HS.

### Impact of Heat Stress on Rumen and Rumen Microbes of Dairy Cows

The appetite center of the hypothalamus is negatively affected by changes in the environmental temperature, resulting in reduced feed intake (Baile and Forbes, 1974). In lactating cows, feed intake starts to decrease at ambient temperatures of 25–26°C and rapidly decreases above 30°C in temperate climate conditions. A decline of up to 40% can occur at 40°C (Rhoads et al., 2013), as well as at 8–10% in buffalo heifers (Hooda and Singh, 2010). Feed intake is an essential source of heat production in ruminants; therefore, reducing feed intake helps to decrease heat production in warm environments (Kadzere et al., 2002). This results in a negative energy balance, which consequently decreases the body condition score and weight of the animal (Das et al., 2016). Moreover, the basic physiological mechanisms of the rumen are altered by high environmental temperatures, which negatively affect ruminants, resulting in health problems and a high risk of metabolic disorders (Nardone et al., 2010; Soriani et al., 2013). HS negatively affects energy metabolism in animals, resulting in reduced production of metabolic heat necessary for normal body temperature maintenance (Kang et al., 2019). It can also reduce rumination, rumen activity, and reticulo-rumen motility, thus affecting the fractional passage rate of digesta in the gastrointestinal tract (Kadzere et al., 2002).

Meanwhile, earlier investigations discovered that HS could reduce ruminal pH and increase the level of lactate in the rumen (Zhao et al., 2019). Greater lactate production decreases the availability of energy, reduces ruminal pH, and inhibits pH-sensitive rumen bacterial growth, which gives rise to subacute ruminal acidosis (SARA), a well-known metabolic disorder that suppresses the production of milk in dairy cows (Russell and Rychlik, 2001; Khafipour et al., 2009; Zhao et al., 2019). Several authors have stated that the decrease in rumination time as a direct effect of heat is one of the reasons for ruminal pH reduction (Moallem et al., 2010; Soriani et al., 2013; Müschner-Siemens et al., 2020). Saliva, which serves as a buffering agent and is essential for isotonic function, decreases due to short rumination time (Meneses et al., 2021). The possible alteration of rumen microbiota might also reduce the ruminal pH. In connection to this, the rumen pH of Holstein heifers decreased when the animals were maintained at environmental temperatures ranging from 20 to 33°C (Tajima et al., 2007). Furthermore, cows with higher ruminal temperature values had an altered rumen microbial population (Yadav et al., 2013; Correia Sales et al., 2021). This includes a reduction in *Fibrobacter succinogenes, Flavonifractor, Prevotella ruminicola, Ruminococcus flavefaciens*, and *Treponema*. The reduction of these rumen bacteria increased the population of lactic acid bacteria because of the amount of substrate suitable for their metabolism. These bacteria produce a large amount of lactate in the rumen, which causes a drastic decrease in ruminal pH (Yadav et al., 2013; Correia Sales et al., 2021). It is known that animals under HS show altered rumen functions, resulting in increased propionate and butyrate production and reduced acetate concentrations (Nonaka et al., 2008). In response, animals consumed minimal amounts of roughage, leading to decreased rumen motility and rumination (Nardone et al., 2010; Soriani et al., 2013) as well as changes in rumen microbial population and pH ranging from 5.82 to 6.03 (Hall, 2009). Thus, HS affects animal health through decreased dry matter intake, reduced saliva production, and changes in digestive functions (Nardone et al., 2010; Soriani et al., 2013). Furthermore, to reduce metabolic heat production, hypofunction of the thyroid gland and effects on animal metabolism patterns are all remarkable results of HS (Helal et al., 2010; Das et al., 2016).

High ambient temperature is a major factor influencing HS, which adversely affects and reduces volatile fatty acid (VFA) production (Nonaka et al., 2008; Yadav et al., 2013; Meneses et al., 2021). Specifically, the acetate to propionate ratio decreases during HS, which results in individual ruminal pH variations, passage rate, and retention time of digestion (Tajima et al., 2007; Nonaka et al., 2008; Yadav et al., 2013; Correia Sales et al., 2021; Meneses et al., 2021). Furthermore, depression of ruminal activity decreases VFA production in the rumen (Kang et al., 2019). Meanwhile, previous studies have observed that plasma lactate levels increase in dairy cows under HS conditions (Tian et al., 2015; Min et al., 2016a). This indicates that a higher concentration of lactate in the rumen could result in increased transport to the blood, which negatively affects the health of the animal (Newbold et al., 2010). Moreover, rumen acetate levels were significantly reduced by HS (Zhao et al., 2019). In connection to this, lactate and acetate are both well-known major metabolites of fiber and soluble carbohydrates. HS significantly changed feed intake, and in particular, more concentrate (rather than forage) is consumed by the animal (Uyeno et al., 2010). Subsequently, a greater proportion of concentrate intake in the diet might be the reason for lactate and acetate changes in the rumen of heat-stressed dairy cows (Zhao et al., 2019). Furthermore, greater VFA metabolism and consumption from arterial blood resulted in a decrease in VFAs during HS conditions (Martz et al., 1971; Kang et al., 2019).

Protein digestion imbalance could be identified if ammonia concentrations were determined, as the increase in ammonia may result in a degraded dietary protein surplus, or even lead to a low degraded carbohydrate concentration in the rumen (Ribeiro et al., 2001). Cows that were fed with isonitrogenated low energy level diet led to a higher N-NH_3_ level and higher rumen degradable protein (RDP), which was due to the silage and inclusion of higher urea levels via supplementation; this result was in contrast with heifers fed with higher energy levels. The accumulation of ammonia as a result of nitrogen compound fermentation when there is an energy source in the substrate for microorganisms could explain this result (Meneses et al., 2021).

Heat stress can affect the rumen microbiome of lactating dairy cows. According to the linear discriminant analysis effect size (LEfSe), five microbial taxa can be used to distinguish normal from heat-stressed Holstein cows, whereas in Jersey cows, twenty-nine such taxa were identified (Kim et al., 2020). Phylum Fibrobacteres, class Fibrobacteria, order Fibrobacterales, family Fibrobacteraceae, and *Arboricola* sp., were enriched in heat-stressed Holstein cows. However, the phyla Fusobacteria, Tenericutes, and Cyanobacteria; Sphingobacteria, Tissierella, Fusobacteria, Mollicutes, Epsilonproteobacteria, and Flavobacteria; Brachyspirales and Mycoplasmatales; one taxon of the family Brachyspiraceae; genera *Staphylococcus* and *Clostridium*; *C. botulinum* sp., *B. cereus* sp., *B. cereus group* sp., and *Xanthomonas arboricola* sp. were enriched in heat-stressed Jersey cows (Kim et al., 2020). Based on the analysis of the bacterial composition in the rumen of Holstein cows using 16S rRNA amplicon sequencing, the relative abundance of members of the phylum Fibrobacteres, specifically the family Fibrobacteraceae, increased significantly under exposure to HS conditions (Kim et al., 2020). One possible reason is that the Fibrobacteres and families under the Fibrobacterales group are known for their higher resistance to heat compared to other ruminal bacteria (Kim et al., 2020). This group is associated with cellulolytic activity, which is common in ruminal microbes (Puniya et al., 2015). The rumen microbial metabolism efficiency is directly related to heat generation in the rumen, which can be indirectly estimated through bacterial growth efficiency (Russell, 1986; Kim et al., 2020). Generally, microorganisms have unique mechanisms of responding to HS, such as adapting a specific favorable temperature and resistance to higher temperatures (Shapiro, 1998).

In contrast, the relative abundance of Actinobacteria, a known acetate-producing bacterium, decreased during HS (Kim et al., 2020). Actinobacteria are classified as gram-positive bacteria capable of metabolizing starch and starch-like polysaccharides and oligosaccharides, which produce lactate and acetate as the major metabolic end-products (Ventura et al., 2007). In previous studies, HS led to a significant increase in the abundance of soluble carbohydrate-utilizing bacteria such as *Streptococcus*, unclassified Enterobacteriaceae, *Ruminobacter*, *Treponema*, and unclassified Bacteroidaceae (Zhao et al., 2019). The major genus of lactate-producing bacteria in the rumen is *Streptococcus* (Nagaraja and Titgemeyer, 2007; Calsamiglia et al., 2012) and the majority of Enterobacteriaceae also produce lactate (Zhao et al., 2019); thus, they potentially precede an increase in lactate concentration and a decrease in rumen pH. An increase in lactate levels and reduced pH is accompanied by an increase in the abundance of *Streptococcus* in the rumen (Wang et al., 2012). Thus, increased production of lactate in the rumen under HS results from an increase in the abundance of lactate-producing bacteria (e.g., *Streptococcus*) (Zhao et al., 2019). Bekele et al. (2011) also stated that *Treponema* is primarily involved in the degradation of pectin and participates in the digestion of concentrates. Furthermore, *Ruminobacter amylophilus* (a representative species of Ruminobacter) shows high starch degradation ability in the rumen (Anderson, 1995). Therefore, the increased abundance of lactate-producing or soluble carbohydrate-digesting bacteria in heat-stressed dairy cows may be attributed to the increase in the dietary concentrate-to-roughage ratio (Zhao et al., 2019). Additionally, HS leads to a decrease in the abundance of acetate-producing *Acetobacter* (Zhao et al., 2019), which can produce acetate by oxidizing sugars (Lyons et al., 2018). Decreased *Acetobacter* abundance is consistent with a decrease in acetate in the rumen fluid (Zhao et al., 2019). The main bacterial taxa that were reported as being affected by HS based on 16S rRNA sequencing are summarized in Table 1.

**TABLE 1:** Affected phyla, class, orders, family, genera, and species during heat stress, obtained through metataxonomic 16S rRNA gene sequencing.

Phylum	Class	Family	Genus	Description (Genus)	Species
Firmicutes	Bacilli	Bacillaceae	*Bacillus*	Transitory bacterium of digestive tract and resistant to heat and cold due to spores (Cutting, 2011); can increase anaerobiosis in digestive tract which enhances growth of Lactobacilli capable of lactic acid production (Souza et al., 2017).	*Bacillus cereus* group
		
			*Streptococcus*	Utilize soluble carbohydrates as an energy source (Zhao et al., 2019); major lactate-producers in the rumen that causes reduction of ruminal pH (Nagaraja and Titgemeyer, 2007; Calsamiglia et al., 2012; Wang et al., 2012)	
	
	Clostridia	Clostridiaceae	*Clostridium*	Increasing dietary concentrate levels significantly increased its relative abundance (Chen et al., 2021); an abundant genus in gastrointestinal tract and some members are distinguished as pathogenic (Dowd et al., 2008; Rajilić-Stojanović and de Vos, 2014); remarkably associated with high-concentrate feeding (Khafipour et al., 2011; Plaizier et al., 2018).	*Clostridium botulinum*
		
		Ruminococcaceae	*Ruminococcus*	Low relative abundance during heat stress is recognized due to its cellulose degradation functions (Correia Sales et al., 2021); specialized amylolytic bacteria responsible for cellulose degradation in the rumen (Ze et al., 2015; Ramos et al., 2021b); several species are capable of fermenting starch thus contributed to the higher abundance during high-concentrate diet period (Klieve et al., 2007; Ramos et al., 2021a)	*Ruminococcaceae* bacterium sp.
					*Ruminococcus bromii*
		
		Lachnospiraceae	Unclassified Lachnospiraceae	Members under this group can produce butyrate which promotes development of epithelial cell and health of gut (Biddle et al., 2013; Meehan and Beiko, 2014; Patra and Kar, 2021)	
	
			*Succiniclasticum*	Abundant in high-concentrate fed cattle, which ferment succinate and convert it to propionate, an essential precursor of glucose in ruminants (Van Gylswyk, 1995; Fernando et al., 2010; Shabat et al., 2016; Ramos et al., 2021b); exhibit an important role in pivotal rumen function as rumen homeostasis index because of reducing sulfate and metabolic flexibility (Sheik et al., 2017)	*Succiniclasticum ruminis*

Bateroidetes	Bacteroidia	Prevotellaceae	*Prevotella*	Due to physiological variability, it can perform different functions in rumen such as digest hemicelluloses, pectinolytic activity, and proteolytic activity (Nagaraja, 2016).	*Prevotella* (genus),
					*Prevotella ruminicola*,
					*Prevotella* sp. ne3005,
					*Prevotella* sp. tc2-28
		
		Bacteroidaceae	*Bacteroides*	Utilize soluble carbohydrates as an energy source (Zhao et al., 2019); more efficient in structural carbohydrates degradation (El-Kaoutari et al., 2013; Ramos et al., 2021a); relative abundance decreased during hot summer (Li et al., 2020)	
	
	Flavobacteria	Flavocateriaceae		No data available yet.	
	
	Sphingobacteria			No data available yet.	

Proteobacteria	Gammaproteobacteria		*Xanthomonas*	Some members has wxacO gene that encodes a protein responsible for lipopolysaccharide biosynthesis (Li and Wang, 2011) that triggers inflammation during high-concentrate subacute ruminal acidosis challenge (Khafipour et al., 2009)	*Xanthomonas arboricola* species
		
		Pseudomonadaceae	*Pseudomonas*	Several species are capable of hydrolyzing cellulose (Lynd et al., 2002; Oyeleke and Okusanmi, 2008; Jin et al., 2016)	
		
			*Ruminobacter*	Utilize soluble carbohydrates as an energy source (Zhao et al., 2019); responsible for degradation of high starch in the rumen (Anderson, 1995); amylolytic group increased in response to heat stress due to rumen acidosis (Li et al., 2014; Baek et al., 2020)	*Ruminobacter amylophilus*

		Enterobacteriaceae		Heat stress increased the abundance of soluble carbohydrate-utilizing bacteria, a known acetate-producers (Zhao et al., 2019).	
	
	Betaproteobacteria	Burkholderiaceae		No data available yet.	
	
	Alphaproteobacteria		*Acetobacter*	Capable of producing acetate by oxidizing sugars (Lyons et al., 2018); relative abundance reduced due to heat stress (Zhao et al., 2019); through taking up of oxygen, it helps to construct an anaerobic environment to enhance the growth of archaea and anaerobic bacteria (Zhao et al., 2019).	

	Epsilonproteobacteria			No data available yet.	

Actinobacteria	Actinobacteria	Streptomycetaceae	*Streptomyces*	Modulate the rumen environment through altering the metabolism of gram-positive bacteria such as cellulolytic, lactate producing, methanogenic, and proteolytic bacteria (Marques and Cooke, 2021).	

Cyanobacteria	Cyanobacteria			No data available yet.	

Fibrobacteres	Fibrobacteria	Fibrobacteraceae		Enriched abundance (phylum to order) is due to its strong heat resistance than other bacteria in the rumen (Kim et al., 2020); this group is associated with cellulolytic activity, a common metabolism of ruminal microbes (Puniya et al., 2015).	

Tenericutes	Mollicutes			No data available yet.	

Spirochaetes			*Treponema*	Predominates the rumen with high energy diets (Bekele et al., 2011; Liu et al., 2014a; Zhao et al., 2019; Correia Sales et al., 2021); species belonging to this genus can utilize polymers as fermentable substrates such as arabinogalactan, pectin, and xylan (Kobayashi, 2006); do not use cellulose as fermentable substrate (Paster and Canale-Parola, 1982; Correia Sales et al., 2021); utilize soluble carbohydrates as an energy source (Zhao et al., 2019); involved in the digestion of concentrates (Bekele et al., 2011).	
	
		Brachyspiraceae		No data available yet.	

It was previously hypothesized that stressor-induced alterations of the gut microbiome lead to physiological and immune alterations (Chen et al., 2018). The same authors concluded that the exposure of animals to HS triggers physiology and immunity, which may be responsible for microbial activities and altered circulation levels of cytokines, supporting the role of the brain-gut axis in dairy cows. HS, rather than heat sensitivity, affects the physiological characteristics, cytokines, and microbial composition of the rumen (Chen et al., 2018). However, it should be noted that the gut microbiome and metabolism can also be indirectly affected by HS. This is because HS can induce changes in several factors, such as reduced dry matter intake, sorting of preferred feed portions, decreased bolus chewing time, and salivary bicarbonate infusion into the rumen, all of which are related to the gut microbiome and metabolism (Zhao et al., 2019). Lastly, changes in the abundance of other microbes, such as anaerobic fungi, archaea, and protozoa, can be induced by HS and require further study (Zhao et al., 2019).

### Effect of Heat Stress on Production, Physiology and Cytokines in Dairy Cows

#### Production and Physiology

A recent study indicated that HS affects body temperature indices and causes significant damage to the thermal homeostasis mechanisms of cows (Meneses et al., 2021). Ocular and rectal temperatures, including pulse and respiratory rates, are altered due to high environmental temperatures (Sharma et al., 2013). The activation responses of thermal receptors, the central nervous system, and sweat gland modification are all physiological responses affected by HS (Collier et al., 2008). Moreover, research has found that ambient temperature affects the heart rate of cattle (Meneses et al., 2021). Their results showed that heat-stressed cows had a higher heart rate (15% increase in the rate of beats per minute), especially when the ambient temperature reached 30°C or higher. The increase in heart rate usually results in the buildup of blood fluidity to the arteries, attempting to increase the dissipation of heat to the environment (Beatty et al., 2006). In the case of respiratory rates, a higher rate is a part of the animal response to increase loss of heat during hot conditions (Meneses et al., 2021). High volume in the alveolar stream is linked to a severe increase in respiratory rate, resulting in high water loss via alkalosis, high excretion of respiratory CO_2_, and respiratory evaporation (Jessen, 2001). Meanwhile, alkalosis resulted in a decrease in CO_2_ and HCO_3_ concentrations and an increase in pH (Jessen, 2001; Mader and Griffin, 2015).

Heat stress reduces DMI, feed conversion efficiency, growth, milk quality and production, and reproductive performance (Jordan, 2003; Brown-Brandl et al., 2005; Rhoads et al., 2009; Wheelock et al., 2010; Bagath et al., 2019). It has been reported that HS can adversely affect and compromise milk yield and immune function (do Amaral et al., 2011; Tao et al., 2012a). Elevated temperature also influenced passive immunity transfer to the calves, which might be linked to reduced absorption of IgG in the intestine after birth. The authors have suggested that HS negatively affects maternal immunoglobulin transfer to colostrum in dairy cows (Nardone et al., 1997; Tao et al., 2012a). Similarly, research shows that exposure to hot conditions during late pregnancy could reduce the quality of colostrum along with decreased concentrations of IgG, IgA, casein, fat, lactalbumin, lactose, and protein (Godden, 2008; Bernabucci et al., 2013). Changes in colostrum composition result in nutritional restriction and mammary blood flow reduction, which impairs IgG and nutrient transfer from the blood to the mammary gland (Godden, 2008).

The negative impacts of HS on domesticated animals threaten animal husbandry and animal production systems, thereby severely damaging the socio-economic status of many countries (St-Pierre et al., 2003; Bernabucci et al., 2010; Dunshea et al., 2013). The dairy industry is severely affected by this due to the high susceptibility of dairy cows to HS (Bernabucci et al., 2014). A substantial decrease in milk production and milk quality is one of the most noticeable production problems associated with HS in lactating dairy cows (Min et al., 2017). Due to the rapidly increasing global temperatures, dairy cows suffering from HS are predicted to be a large-scale problem in the future (Zhao et al., 2019).

Decreased feed intake due to HS was previously assumed to be directly responsible for reduced milk yields (Fuquay, 1981; Collier et al., 1982); however, evidence showed that feed intake reduction only contributed to approximately 35% of the HS-induced decrease in milk production yield (Rhoads et al., 2009). Further research suggested that up to 50% of HS-induced feed intake reduction could affect lactation (Sammad et al., 2020). Rather, other intake-independent alterations induced by HS, particularly those related to nutrient partitioning, may be associated with changes in post-absorptive glucose and lipid metabolism changes (Wheelock et al., 2010).

**FIGURE 1:**
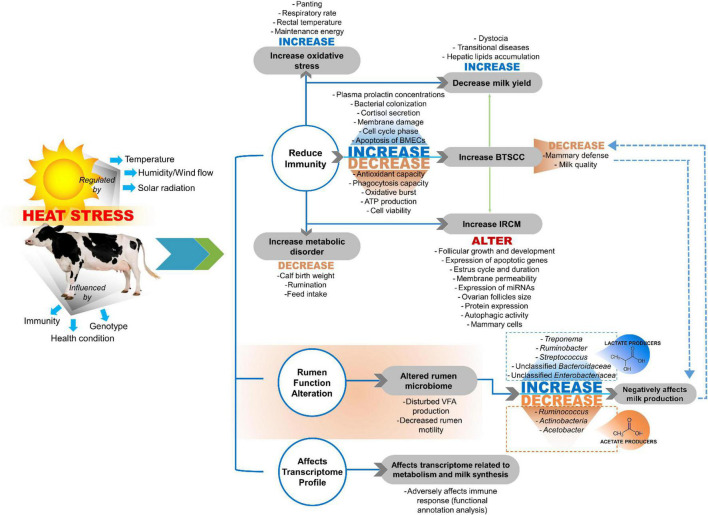
Impact of heat stress on dairy production. BTSCC, bulk tank somatic cell count; IRCM, incidence rate of clinical mastitis (Kim et al., 2020; Rakib et al., 2020; Sammad et al., 2020).

The adverse effects of HS are influenced at the cow level by genotype, health condition, and immunity. Moreover, the adverse effects of HS are influenced by atmospheric temperature, humidity, wind flow, and solar radiation (Figure 1; Pragna et al., 2017). Altered normal physiological functions of dairy cows due to HS cause a higher incidence of udder health issues, especially during the summer season (Turk et al., 2015). In addition, the high ambient temperature and high relative humidity during summer enhance the growth of microorganisms responsible for mammary gland infections, which challenges the mammary defense capacity and induces bacterial colonization of the gland (Barkema et al., 1998; Olde Riekerink et al., 2007; Gao et al., 2017; Fan et al., 2019; Rakib et al., 2020). Thus, understanding the mechanisms by which HS negatively affects dairy cows is essential for developing proper strategies for mammary gland health maintenance during the HS period (Rakib et al., 2020).

#### Arginine

One of the potential mechanisms of amino acid arginine in protecting intestinal integrity during HS has recently been identified (Varasteh et al., 2018; Lian et al., 2020). This mechanism may be related to HSP70 expression enrichment. Similar results found that supplementation with arginine significantly increased and upregulated the expression levels of HSP70 and HSP90 (Xia et al., 2019). Preventing the activation of conventional protein kinase C (cPKC) by reducing the myosin light chain (MLC) protein phosphorylation of the actin cytoskeleton is a possible mechanism of HSP70 which lessens the epithelial barrier dysfunction during stress condition (Yang et al., 2007; Zuhl et al., 2014). Furthermore, arginine enhanced the proliferation of cells, which decreased apoptosis under HS conditions through high expression of growth arrest and DNA damage-inducible beta (Gadd45b), growth factor receptor bound protein 7 (Grb7), marker of proliferation Ki-67 (Mki67), and stress-induced phosphoprotein 1 (Stip1). It also strengthens intestinal mucosal immunity and gut barrier functions, minimizes oxidative damage, and initiates the proliferation of enterocytes (Lian et al., 2020). Through the activation of mammalian target of rapamycin (mTOR), focal adhesion kinase (FAK), and nitric oxide (NO) cascades, arginine can contribute to mucosal repair of the intestinal epithelium and healing of wounds (Grishin et al., 2016). Physiologically, arginine has an essential function in metabolic synthesis pathways, such as the production of polyamine and NO. These are important in enterocytes and multiple cellular signaling pathways, including blood flow, healing processes, intestinal immunity, and protein synthesis (Morris, 2004; Rhoads et al., 2008). Previous research revealed that arginine protects cells by inhibiting HS-induced changes in mitochondrial membrane potential, expression of apoptosis-related genes, antioxidant enzyme regulation, and inflammation (Dou et al., 2021). In addition, the decrease in damage in bovine intestinal epithelial cells by reducing oxidative stress and inflammation caused by HS indicates that arginine could be effective in protecting cows from the harmful intestinal effects of HS (Dou et al., 2021). These data imply that arginine protects intestinal tissues against the negative effects of HS (Xia et al., 2019).

#### Cytokines

Heat stress has been recognized to adversely affect the immune system through humoral and mediated immune responses. By triggering the hypothalamic-pituitary-adrenal (HPA) axis and increasing peripheral glucocorticoid levels, HS suppresses the production and distribution of cytokines. Additionally, it stimulates an increase in the concentration of blood cortisol, which inhibits the synthesis of cytokines such as tumor necrosis factor-α (TNF-α), interleukin-4 (IL-4), IL-5, IL-6, IL-12, and interferon γ (IFN-γ) (Bagath et al., 2019). These findings were similar to those of previous studies, wherein the plasma circulation levels of cytokines such as interleukin-1β (IL-1β), IL-6, IFN-γ, and TNF-α were affected and were found to be higher in heat-stressed cows (Chen et al., 2018). This implies a possible inflammatory condition that verifies the fact that oxidative stress can cause inflammation (Brook, 2008; Mohamed Ibrahim, 2015; Min et al., 2016b). One of the mechanisms by which cattle defend against the detrimental effects of environmental challenges is the immune response (Stephanou and Latchman, 2011). During HS, the signal transduction pathways are stimulated, which leads to the alteration of gene expression of immune cell mediators, promoting cytokine activity due to heat shock response activation. The effector functions and the development of T-helper 1 (Th1) and T-helper 2 (Th2) cell responses are due to distinct cytokine patterns (Bagath et al., 2019). For instance, cell-mediated immunity and pro-inflammatory responses are activated by Th1 cells, while activation of humoral immunity and anti-inflammatory cell responses are activated by Th2 cells (Hendrix and Nitsch, 2007). An important component involved in the regulation of the balance between these cytokines in animals is the selective production of Th1 cytokines (IFN-γ and IL-12), as well as the selective production of Th2 cytokines (IL-10, IL-4, and IL-13) (Park et al., 2005). A good example of this is the downregulation of Th1 cytokines and upregulation of Th2 cytokines, which leads to the suppression of cell-mediated immunity, resulting in hyperthermia CMI (Webster et al., 2002; Lacetera et al., 2005). HS damages the cellular immune response through the accumulation of cortisol, which binds to DNA, inhibiting the expression of genes involved in the activation and production of T-cells and cytokines, respectively (Caroprese et al., 2012; Sgorlon et al., 2012). Decreased activity of phagocytic cells and altered function of lymphocytes are the result of the anti-inflammatory properties of corticosteroids (Caroprese et al., 2009). Furthermore, the altered ratio of Th1 and Th2 affects the immune system of animals due to HS. The immune system is being challenged to maintain homeostasis, but a shift might result in disease susceptibility and become immunocompromised (Elenkov et al., 2000). Thus, maintenance of the Th1:Th2 balance is crucial to minimize the adverse effects of immunological challenges during HS conditions (Bagath et al., 2019).

#### White Blood Cells and Somatic Cells

Common genes that showed a fold change in similar directions were differentially expressed both in peripheral white blood cells (WBCs) and milk somatic cells during the heat challenge (Garner et al., 2020). The upregulated HSP transcripts responding to heat challenge were expressed in both cell types. These include HSPA4, HSPA6, HSPA1A, HSPA1L (HSP70), HSP90AB1 (HSP90), HSPH1 (HSP 105), and AHSA1 and AHSA2, both of which are activators of HSP90 (Garner et al., 2020). In this connection, high HSP expression is well-known as a cellular response to HS. The cellular response to stress occurs at the systemic level and locally in the mammary gland. This can be explained by the upregulation of HSP genes, which indicates that the peripheral WBCs and somatic cells in the milk respond to HS *in vivo*. Previous researchers have found that WBC of heat-stressed cattle increased by 21–26% due to thyromolymphatic involution or erythrocyte destruction (Kamwanja et al., 1994; Hu et al., 2016a; Bagath et al., 2019).

### Impact of Heat Stress on Transcriptome and Proteome Profile

#### Transcriptome Profile

Analysis of the transcriptome obtained from peripheral WBCs and milk somatic cells revealed that certain genes, particularly those involved in cellular stress response, apoptosis, oxidative stress, and glucose metabolism, were differentially expressed (Garner et al., 2020). Genes associated with inflammation, lipid metabolism, carbohydrate metabolism, and the cardiovascular system were differentially expressed at a significant level in the peripheral WBC between heat-challenged cows and thermoneutral control, indicating that heat challenge could induce alterations in gene expression. In addition, the transcriptome analysis of the peripheral WBCs uncovered certain complex changes in the gene expression patterns that are related to major alterations in the metabolism of challenged cows, including Bradykinin receptor B1 (BDKRB1) and 3-hydroxybutyrate dehydrogenase 2 (BDH2) (Garner et al., 2020). BDKRB1, which was observed to be the most upregulated gene, indicated that there was an acceleration in the inflammation rate during heat challenge, which is transiently induced by injury in the tissue or by inflammation (Raslan et al., 2010). WBCs increased by 21–26% in heat-stressed cattle due to thyromolymphatic involution or erythrocyte destruction (Abdel-Samee, 1987; Bagath et al., 2019).

Additionally, the observed downregulation of BDH2 further supported that inflammation, including those associated with BDKRB1, such as pro-inflammatory cytokine release, immune cell flux, and increased vascular permeability, was a key biological response to HS. This change in the expression of BDH2 can be implicated in the modulation of the iron-limiting innate immune response (Zughaier et al., 2014). These two genes indicate that inflammation is one of the major biological pathways affected in peripheral WBCs by heat challenge is inflammation (Garner et al., 2020). HS downregulates the expression of main milk protein-encoding genes and several key genes related to the regulation of protein synthesis, and amino acid and glucose transport (Gao et al., 2019).

Furthermore, the metabolic activity of the mammary tissue, especially carbohydrate and lipid metabolism, generally decreased, while immune activation and inflammation increased, as analyzed based on transcriptomic data (Gao et al., 2019). The major role of the inflammatory response of insulin-like growth factor 1 (IGF-1), interferon gamma (IFNG), S100 calcium binding protein A8 (S100A8), S100 calcium binding protein A9 (S100A9), and tumor necrosis factor (TNF) to induce or control the inflammatory response with the aid of nuclear factor kappa-light-chain-enhancer of activated B cells (NF-κB) in the process of immunoactivation was revealed through network analysis (Gao et al., 2019). NF-κB plays an important role in cellular responses to stimuli such as bacterial or viral antigens, cytokines, free radicals, heavy metals, oxidized LDL, stress, and UV irradiation (Brasier, 2006; Bu et al., 2017).

Biological processes such as chaperone-dependent refolding of proteins, HSP binding activity, and immune response were found to be affected by HS through gene ontology functional analysis (Yue et al., 2020). During HS, activation of HSF and increased expression of HSPs are the usual responses at the cellular level to cope with homeostasis (Collier et al., 2008). Evidently, HSF (a transcription factor family) has been determined to be the primary responder during HS (Helfand et al., 2003; Page et al., 2006). On the other hand, HSPs are considered potential markers for environmental adaptation of animals and are correlated with resistance to stress (Feder and Hofmann, 1999). Based on the molecular weight and biological functions of livestock species, HSP70 and HSP90 are the most abundant and are primarily correlated with the development of heat tolerance (Belhadj Slimen et al., 2016). Among these, the HSP70 gene is considered a reliable biological marker to measure HS response (Bharati et al., 2017), because it is involved in cellular protection against acute HS or physiological stimuli (Maloyan et al., 1999). Exposure to HS acts as a stimulus that leads to dissociation of the HSF1 monomer, to which under unstressed conditions, is bound to the HSP in the cytoplasmic matrix. This then leads to the binding of the dissociated HSF1 with other HSF monomers that undergo trimerization prior to translocation to the nucleus (Archana, 2017).

Heat stress affects gene transcription, which triggers the binding of the homotrimeric HSF to HS elements in the nucleus and hyperphosphorylation, resulting in increased expression of HSP mRNA (Collier et al., 2008). HSF1, previously associated with HSP regulation, is associated with carbohydrate metabolism, cytoskeleton, transport, and ubiquitination during HS (Page et al., 2006). The majority of the top-affected pathways were associated with immune responses based on Kyoto Encyclopedia of Genes and Genomes (KEGG) enrichment analysis. The expression of bovine lymphocyte antigen (BoLA) and histocompatibility complex, class II, DRB3 (BoLA-DRB3) was downregulated during HS; however, the HSP 90 kDa beta I (HSP90B1) and heat shock 70 kDa protein 1A were upregulated. Moreover, mammary gland tissue of cows under HS conditions increased the expression of cytosolic arginine sensor for mTORC1 subunits 1 (CASTOR1) and 2 (CASTOR2), and phosphorylation of mammalian target of rapamycin, which decreased the phosphorylation of Janus kinase-2, a known signal transducer and activator of transcription factor-5. Consequently, HS has historically negatively affected the immune function of dairy cows, as seen in DMI, milk yield, casein gene expression, and genes and pathways identified through functional annotation analysis (Yue et al., 2020).

#### Proteome Profile

Environmental HS adversely affects biochemical pathways, immune and inflammatory responses, physiological and performance traits, and protein profiles in dairy cattle (Sheikh et al., 2017; Maibam et al., 2018; Skibiel et al., 2018; Abdelnour et al., 2019). Various studies on gene expression profiles have suggested that transcription and translation of RNA in mammary epithelial cells can be inhibited by HS (Li et al., 2017). Gene expression profiling showed both upregulation of genes associated with protein repair and stress response, and down-regulated genes associated with cellular metabolism, mammary epithelial cell-specific biosynthesis function, and morphogenesis once bovine mammary epithelial cells were exposed to high temperatures (Collier et al., 2006, 2008). Most mammary cell proteomic studies in bovines have sequenced pooled protein samples from various pathological conditions and lactation periods (Huang et al., 2014; Zhang et al., 2015). High environmental temperatures negatively affect the nutritional status and metabolism of livestock species (Abdelnour et al., 2019). Consequently, HS altered the proteomic profile of dairy cows during the transition period (Table 2).

**TABLE 2:** Impact of heat stress on proteomic profile of dairy cows.

Affected proteins	Description (heat stress indicators)	References
(1) Heat shock proteins (HSP) family	• Many studies have shown that heat stress condition increased the gene expression of HSPs and secreted proteins.	Gaughan et al., 2013; Deb et al., 2014; Liu et al., 2014b; Min et al., 2015
	• HSP family is associated with protein denaturation prevention, and repairing unstable proteins produced during heat stress, thus plays a cytoprotective role and interact with diverse types of cellular proteins.	Garrido et al., 2001; Silver and Noble, 2012; Velichko et al., 2013
	• Previous research showed that levels of HSP70 increased during the initial period and progressively decreased in mammary epithelial cells when exposed to acute heat stress.	Collier et al., 2006; Wang et al., 2012; Hu et al., 2016a,b
	• HSPs expression is associated to kinetics of thermotolerance acquisition, decay, and maintenance.	Landry et al., 1982; Ma et al., 2019
	• Exposure to extreme heat stress in dairy cows increased the expression of HSP, thus its synthesis may reduce the availability of circulating amino acid essential for milk protein synthesis.	Collier et al., 2008; Cowley et al., 2015
(2) Blood amino acids profile	• Concentrations of total alanine, amino acids, aspartate, glutathione, glycine, and threonine significantly increased.	Guo et al., 2018
	• Metabolism of nitrogen disruption and incentive nitrogenous repartition. • Milk protein percentage declining. • Increase urea level in milk.	Cowley et al., 2015
(3) Liver proteomics	• Altered liver proteomic profile. • Heat stressed cows have reduced ATP synthesis, shifts in the precursor supply for gluconeogenesis, greater oxidative stress, and liver hepatic lipids accumulation which might contribute to fatty liver disease. • Impaired mitochondrial function and altered carbohydrate, lipid, and amino acid metabolism in the liver.	Skibiel et al., 2018
(a) Cytochrome b-c1 complex subunit 6	• Essential component for cytochrome c1 and cytochrome complex. • Reduced cytochrome c oxidase complex activity reduces production of ATP and supplying glycolysis for the synthesis of ATP.	Yasuo and Shigeo, 1990; Li et al., 2006
(b) NADH dehydrogenase [ubiquinone] 1 beta subcomplex subunit 6	• One of core subunits of complex I which is related to the activity and stability of complex I. • Oxidative stress will lead to impairment of complex I.	Leman et al., 2015
	• Heat-stressed cows had decreasing various subunits of NADH dehydrogenase complex.	Ma et al., 2019
(4) Interleukin	• Heat stress resulted to reduced milk production throughout the lactation period, and cattle are more susceptible to metabolic disorders.	Kadzere et al., 2002; Bernabucci et al., 2010; Tao and Dahl, 2013
	• Mammary gland remodeling and hepatic lipid metabolism alteration.	do Amaral et al., 2009, 2011; Tao et al., 2011
(5) Oxidative stress markers	• Significant increase in level of reactive oxygen species (ROS) synthesis.	Maibam et al., 2018
	• Excessive production of ROS could lead to disruption of anti-oxidant defense enzymes which produces oxidative stress in ruminants.	Celi, 2011
	• Complications of heat stress tend to increase due to excessive synthesis of ROS which diminishes anti-oxidant defense, thus resulting to oxidative injury.	Bernabucci et al., 2002; Di Trana et al., 2006; Chauhan et al., 2014b
(6) Inflammatory gene expression [Nuclear factor kappa B (NF-κB) and tissue tumor necrosis factor α (TNF-α)]	• Heat stress stimulates several physicochemical responses such as upregulation of inflammatory genes (NF-κB and TNF-α).	Guo et al., 2018; Maibam et al., 2018
	• Involved in inflammation which is promoted due to oxidative injury caused by heat stress.	Chauhan et al., 2014a
	• Main regulatory of inflammatory signaling which plays a key role in proinflammatory cytokines synthesis.	Montilla et al., 2014
(7) Antioxidant indices		
(a) Glutathione peroxidases (GPx)	• GPx enzymes are critical defense enzymes and have vital functions in cell protection against oxidative injury.	Hefnawy and Tórtora-Pérez, 2010; Chauhan et al., 2014a,b
	• Significant increase in levels of GPx and GRx in the skin of cattle during thermal environmental stress in cattle.	Maibam et al., 2018
(b) Malondialdehyde (MDA)	• Sensitive biomarker of oxidative stress, thus the increase in MDA level due to heat stress strongly affected the animals and contribute adverse impact on immune responses.	Abdelnour et al., 2019
	• Reduction of level of MDA which subsequently disrupting intestinal cell function and structure.	Chauhan et al., 2014b; Rathwa et al., 2017; Guo et al., 2018; Maibam et al., 2018
	• Enhanced accumulation of MDA inhibits the activity of antioxidant enzymes in the mitochondria of cells.	Mujahid et al., 2007
(c) Superoxide dismutase (SOD)	• One of the most important cellular defense enzymes because it can enhance production of superoxide free radicals in the mitochondrial electron transport chain and prevent mitochondrial membranes oxidative damages.	Matés et al., 1999
	• Heat stress significantly alters cellular antioxidant machinery, structure, and metabolism of carbohydrates and skeletal muscle.	Cruzen et al., 2015
(8) Chitinase-3-like protein 1 (CHI3L1)	• Increase in mammary tissue of heat stressed cows. • It is a minor milk protein in mammary sections that mediates mammary tissue differentiation and remodeling. • Increase in CHI3L1 negatively associated with lactogenesis in mammary gland which resulted to decrease in milk yield of heat-stressed cows.	Rejman and Hurley, 1988; Stiening et al., 2008; Ma et al., 2019
(9) Mitochondrial malate dehydrogenase (MDH1 and MDH2)	• Decrease expression in heat-stressed cows. • Function as key enzymes in the tricarboxylic acid cycle (TCA) responsible for energy metabolism through aerobic respiration. • Heat treatment inhibits biosynthesis of nucleic acids and proteins.	DeBerardinis et al., 2008; Rhoads et al., 2013; Tian et al., 2015
(10) Fatty acid synthase (FASN)	• Enzyme responsible for fatty acid biosynthesis in mammary gland.	Bionaz and Loor, 2008; Qi et al., 2014
	• High expression of FASN means that it plays an essential role for fatty acid synthesis, however it has significantly lower expression in heat-treated bovine mammary epithelial cell which suggests that fatty acid synthesis might affected by heat stress.	Zhu et al., 2014; Li et al., 2017

### Effect of Heat Stress on Rumen and Blood Metabolites of Dairy Cows

Heat stress affects different metabolic processes in ruminants, especially in dairy cows, with respect to rumen fermentation functions, blood parameters and metabolites, and shifts in metabolic pathways. Ruminal pH is lower in cattle under HS because of the high concentrations of lactic acid (Yadav et al., 2013). Furthermore, the consumption of less feed and consequent reduced rumination results in reduced amounts of buffer substances entering the rumen, which may explain the decrease in ruminal pH (Sammad et al., 2020). Earlier research found that ruminants under HS showed significantly decreased concentrations of ammonia nitrogen (NH_3_-N) (Cai et al., 2019). This suggests that HS may affect rumen fermentation, digestion, and metabolism of dietary proteins and other nitrogenous compounds (Cai et al., 2019).

Heat stress decreases the proportion of acetate and increases the proportion of butyrate in the rumen (Klieve et al., 2003; Cai et al., 2019). The decrease in acetate might be due to a decrease in *Acetobacter* abundance (Zhao et al., 2019), and the concentrations of total VFAs and propionate in the rumen of heat-stressed animals were significantly decreased (Cai et al., 2019). However during HS, concentrate intake was significantly increased, resulting in increased production of propionate (Uyeno et al., 2010). Changes in the concentrations of VFAs in the rumen may also be due to increased water intake and higher water content in the rumen (Schneider et al., 1988; Cai et al., 2019). In addition, decreased feed intake and changes in the amount and/or activity of microorganisms in the rumen are important factors affecting VFA production (Klieve et al., 2003; Tajima et al., 2007). Although bacterial communities are the most abundant and metabolically diverse in the rumen, ciliate protozoa can contribute to half of the rumen microbial biomass, thus strongly affecting the overall production of VFAs (Newbold et al., 2015; Lyons et al., 2018).

Heat stress also causes changes in blood parameters and metabolites in lactating dairy cattle. Serum pathway analysis revealed that the phenylalanine metabolism pathways, tyrosine, and tryptophan biosynthesis were downregulated (Jo et al., 2021). Phenylalanine, tryptophan, and tyrosine are the aromatic amino acids responsible for protein synthesis. These amino acids play regulatory functions during HS by a co-expression network (Parthasarathy et al., 2018; Jo et al., 2021). The release of catecholamine and glucocorticoid hormones is induced during HS, which typically promotes adipocyte lipolysis and NEFA mobilization (Collier et al., 1982; Cincović et al., 2011). Glucose and NEFA concentrations show daytime-dependent changes in dairy cows exposed to HS induced by solar radiation, causing a decrease in glucose concentration and an increase in NEFA concentrations (Cincović et al., 2011). Reduced feed intake, altered gluconeogenesis, and increased insulin concentrations in the body are the main reasons for the decrease in glucose concentrations (Abeni et al., 2007; Bernabucci et al., 2010; O’Brien et al., 2010). Higher NEFA concentrations are associated with intense subcutaneous lipid mobilization (Vitali et al., 2009; Bernabucci et al., 2010; Cincović et al., 2011). NEFA is released into the bloodstream due to lipolysis and can act as an alternative source of energy (LeRoy Hahn et al., 2013). The mobilization of body fat, which can be used for energy or milk fat synthesis, may lead to the formation of ketone bodies such as acetoacetate, acetone, and beta-hydroxybutyrate (Drackley et al., 1991; Peterson et al., 2012; Tian et al., 2015). Additionally, the concentrations of acetoacetate decarboxylase and 3-hydroxybutyrate dehydrogenase enzymes, which are involved in ketone body production pathways, were significantly higher during HS (Tian et al., 2015). Concentrations of circulating cortisol, norepinephrine, and epinephrine are increased during HS, suggesting catabolic signals that stimulate lipolysis and adipose triglyceride mobilization (Wheelock et al., 2010; Tian et al., 2015).

Consequently, serum concentrations of BUN are higher in animals under HS, which can be attributed to the impact of heat on protein metabolism and amino acid balance such that the absorptive function of the rumen epithelium decreases along with the amount of BUN reabsorbed from the blood, causing BUN to accumulate in the blood (Cai et al., 2019). Moreover, heat-stressed cows and heifers showed increased plasma urea nitrogen levels compared to animals under thermally neutral controls (Bernabucci et al., 2010). BUN can either originate from the inefficient incorporation of rumen ammonia into microbial proteins or from hepatic deamination of amino acids mobilized in skeletal muscles (Bernabucci et al., 2010). Blood mineral metabolites are also affected by HS. Phosphorus and calcium retention, and their concentrations, decrease to maintain blood mineral balance in response to potassium loss caused by increased sweating (Kang et al., 2017). In addition, reduced concentrations of blood Mg occur due to increased utilization of Mg for lipolytic enzymes and decreased Mg transport through the rumen (Calamari et al., 2007).

Moreover, total protein concentrations in serum decrease significantly during HS due to decreased protein intake and utilization (Cai et al., 2019). Plasma creatinine increases during HS, suggesting the mobilization of muscle proteins with subsequent creatinine delivery in the plasma. Plasma creatinine is used as a marker of muscle mass, and increased creatinine concentrations during HS may be due to increased muscle protein mobilization (Abeni et al., 2007). The concentration of blood aspartate aminotransferase (AST) is also affected by HS. Blood AST activity is very important because it acts as a catalyst in the metabolism of amino acids and carbohydrates (Filipejová and Kovacik, 2009). Changes in the levels of this metabolite in the blood can be a consequence of its increased activity in cells or damage to the cell structure (Milinković-Tur et al., 2005). AST is a common enzyme in many tissues and organs, with particularly high levels of activity in the liver (Zimmerman et al., 1968), and increased AST activity in serum is an indicator of liver damage (Cincović et al., 2011). Previous studies found increased bilirubin concentrations in the blood serum of heat-stressed animals, which indicated a decreased excretory capacity of the liver (Cincović et al., 2011).

During HS, liver function is altered, and health problems occur due to nutritional and metabolic acclimation. In particular, due to increased maintenance requirements regarding thermoregulation and reduced feed intake, summer transition dairy cows are at a higher risk of liver lipidosis (Basiricò et al., 2009). Increased liver lipidosis probably compromises liver function, and heat-stressed cattle show reduced albumin secretion and liver enzyme activities (Bernabucci et al., 2002). Reduced plasma cholesterol concentrations occur in combination with lower concentrations of triglycerides during HS, which may be due to an increase in lipid utilization by peripheral tissues (Abeni et al., 2007).

## Alleviation Strategies to Minimize Heat Stress

As discussed earlier, HS is the main factor that negatively affects the production performance, physiology, metabolism, and gut microbiome of lactating dairy cows. Thus, mitigation strategies are required to alleviate the severity of the effects of HS on dairy production (Abbas et al., 2020). These mitigation strategies are categorized into two general approaches: short- and long-term strategies (Table 3), which include nutritional management, environmental modification, and genetic selection of heat-tolerant cows through selective breeding programs (Sammad et al., 2020).

**TABLE 3:** Short- and long-term strategies for alleviation of heat stress in dairy cattle.

Strategies	Management	System	Definition	Example	Impact	References
Short term	Nutritional management	Feeding system	Reformulation to account for reduced DMI, greater nutrient requirements during heat stress, dietary heat increment, and avoiding nutrient excesses.	• Changes in macro and micronutrient composition. Increasing energy and crude protein supply in feed. • RDP and RUP reduction • Dietary bypass fats	Prevent nutrient deficiencies, compensate the reduced feed intake, maintain milk protein synthesis and limit their catabolism, and increased milk yield.	West, 2003; Renaudeau et al., 2012; Das et al., 2016; Kaufman et al., 2018; Sammad et al., 2020
		
		Supplementation	Maintain water balance, nutrients and electrolytes intake and/or to satisfy the special needs during heat stress such as vitamins and minerals.	Biological supplements • Dietary yeasts –*Aspergillus oryzae* –*Saccharomyces cerevisiae* Chemical supplements • Fermentates • Betaine • Dietary cation anion difference • Propionate supplementation • Multivitamins like Vitamin B complex, Vitamin C, Vitamin E, Niacin and Nicotinic Acid • Mineral supplementation such as Mn, Zn, Mo, P, and Se • Ionophores and monensin	Enhance the constrained gastrointestinal tract and the metabolic status of dairy cows, improve energy metabolism status and milk yield, improve the immune system by maintaining general health, and positive effect on production parameters under heat stress.	(Gomez-Alarcon et al., 1991; Bruno et al., 2009) – Biological supplementation (Erdman, 1988; Di Costanzo et al., 1997; Melendez et al., 2003; Renaudeau et al., 2012; Barreras et al., 2013; Bicalho et al., 2014; Wang et al., 2019; Abbas et al., 2020; Sammad et al., 2020) – Chemical supplementation

Long term	Genetic selection	Breeding selection	Classification of heat-tolerant animals within the herd of high-producing animals is especially useful through recordings and allied phenotypes.	• Selection through breeding by considering the anatomical and morphological characteristic of the cattle such as slick hair, white coat color, and low coat density.	Identification and selection of heat-tolerant cattle that will still produce well under such stress.	Amamou et al., 2019; Carabaño et al., 2019; Sammad et al., 2020
	
	Environmental modification	Cooling system	Heat stress measurement and applying proper cooling-facilitative measures to the cows.	• Provision of house or shade (together with feed and water). • Evaporative cooling with water in the form of fog, mist, or sprinkling with natural or forced air movement. • cooling ponds	Reduce the heat acquisition and promote heat dissipation by enhancing evaporation, lower rectal temperature, and respiratory rate, and increasing feed intake and efficiency.	Tao et al., 2012b; Wang et al., 2020

### Short-Term Strategies to Minimize Heat Stress

Providing cool clean drinking water, adjusting feed ingredients, improving feeding and husbandry practices, and the use of mineral and vitamin supplements have been helpful in alleviating the negative effects of HS (Sammad et al., 2020). With increasing ambient temperature, water intake may increase by as much as 50% during periods of HS (Vermunt, 2010). Therefore, providing dairy cattle with cool drinking water is necessary to compensate for increased losses from sweating and increased respiration rates (Vermunt, 2010) and may also lower body temperature. Increasing the amount of energy-rich rations, such as by providing additional concentrate, is necessary to compensate for the reduced intake caused by HS and to counteract the high production of bodily heat caused by feeding forages (Sammad et al., 2020). Increasing dietary energy density is very important (Drackley et al., 1991), however, this strategy should be implemented with caution as such diet alterations may be associated with a lower rumen pH and acidosis (Sammad et al., 2020).

To increase the use of amino acids, it is necessary to maintain milk protein synthesis and limit milk protein catabolism, as well as to reduce rumen degradable (RDP) and non-degradable proteins in cattle exposed to warm temperatures (Kaufman et al., 2018). Reduction of RDP and RUP decreases the supply of metabolizable protein, which causes a decrease in plasma insulin concentrations and consumption of circulating AA in insulin-sensitive tissues (Kaufman et al., 2018). Thus, the RDP and RUP reduction response probably controlled the consumption of amino acids (AA) in peripheral tissue by reducing substrate availability while sustaining the synthesis of milk protein. In contrast, propionate supplementation improves the metabolism status and milk yield in transition cows (Melendez et al., 2003). Propionate is predominantly converted to glucose at a conversion proportion exceeding 30% in the rumen (Leng et al., 1967). Feed consisting of ionophores and monensin stabilized and favored feed intake and feed efficiency, respectively, and altered rumen fermentation parameters by increasing propionate production (Baumgard et al., 2011; Barreras et al., 2013). Monensin, a known rumen modifier, enhances propionate production, which is essential for gluconeogenesis in ruminants, and reduces methane synthesis (Huntington, 1989). The key mechanism by which monensin enhances the feed efficiency of growing and lactating ruminants is by increasing carbon conservation during fermentation (Barreras et al., 2013).

In addition to crude protein (CP) supplementation, a dietary cation–anion difference (DCAD) is recommended (Wildman et al., 2007). During HS, DCAD-supplemented dairy cows had an increased essential amino acid blood concentration, which reduces the requirement for degradation of amino acids to maintain the acid-base balance, sharing AA for other uses such as growth and production of milk (Wildman et al., 2007). Due to their buffering properties in the rumen, dietary bicarbonate (HCO_3_) may also help (Erdman, 1988). HS could lead to rumen acidosis because of an increase in concentrate intake, and reduced feed intake resulted in reduced salivation, which provided buffer to acidotic dairy cows. Thus, dietary bicarbonate supplementation is essential for maintaining a favorable rumen environment through resistance to ruminal pH changes (Erdman, 1988).

Niacin (generically called nicotinic acid) reduces the effects of HS and improves metabolism in lactating dairy cows (Di Costanzo et al., 1997). It stimulates vasodilatory reactions that might be advantageous for heat-stressed dairy cows. Internal and peripheral vasodilation may boost the transfer of heat from the core to skin sites and create a temperature gradient that favors the loss of heat from the skin to the environment (Di Costanzo et al., 1997).

Important options to prevent the adverse effects of HS include dietary yeast and fungal supplementation, such as *Saccharomyces cerevisiae* and *Aspergillus oryzae*, respectively (Sammad et al., 2020). In addition, it has been suggested that feeding fungal cultures such as *A. oryzae* might enhance rectal temperature and respiration rate as visible signs of heat-stressed lactating dairy cows exposed to high ambient temperature (Huber et al., 1994). Several studies showed that feeding fungal cultures could decrease the respiration rate and rectal temperature of heat-stressed dairy cows (Huber et al., 1994). Moreover, feeding of *S. cerevisiae* can alter the rectal temperatures of dairy cows during HS (Bruno et al., 2009).

For cows that were not subjected to HS in the summer, 15.3% dietary CP and rumen bypass fats were shown to help maintain milk yield (Chan et al., 1997; Arieli et al., 2004). Diets containing 15.3% CP with 35% rumen-undegradable protein (RUP) (5.4%, DM), and a suitable ratio of rumen-degradable OM (RDOM) to rumen-degradable CP (RDP) might be sufficient for the maintenance of production of heat-stressed dairy cows that produce 29–38 kg of milk per day (Arieli et al., 2004). This finding is in accordance with the NRC (2000) recommendation of 15.2% CP diets with 5.5% RUP for non-heat-stressed dairy cows that produce 35 kg of milk per day. Energy density requires high fat and low heat increment; thus, fat in the diet of heat-stressed dairy cows should give rise to higher concentrations of energy without increasing the animal body temperature or compromising ruminal fermentation, resulting in high milk yield and better lactation endurance (Chan et al., 1997). Palm oil supplementation increases dry matter intake and reduces the adverse effects of HS (Garcia-Ascolani et al., 2016). According to one study, supplementation of dietary fat up to 5% of lactating cows’ ration supports energetic metabolism (Palmquist and Jenkins, 1980). Supplementation of conjugated linoleic acids improves the negative energy balance during HS, but at the same time, milk fat depression may occur (Sammad et al., 2020). Lipoic acid can exert protective effects to energy metabolism (Diesel et al., 2007), thus lipoic acid supplementation may help heat-stressed animals (Rhoads et al., 2013).

During HS, several metabolic disorders such as ketosis, acidosis, laminitis, hypovolemia, and hypoxia may occur in affected animals. Linoleic acid is a supplement that exhibits promising effects in preventing and/or treating diseases that may occur during HS is linoleic acid, as it can act as a direct binding activator of insulin receptor, in addition to its potent antioxidant potential (Diesel et al., 2007). On the other hand, trace minerals such as Mn, Zn, Mo, P, and Se enhance metabolic status and improve the general health of dairy cows (Bicalho et al., 2014). In addition, vitamins such as B-complex, ascorbic acid, vitamin E (tocopherol), niacin, and nicotinic acid were assessed and found to protect the rumen during HS to be helpful (Rungruang et al., 2014). Several biochemical processes, such as immune response, cell replication, skeletal development, and reproductive performance, require essential trace minerals to function normally (Bicalho et al., 2014). However, a previous study conducted at the University of Arizona demonstrated that, during summer, a 12-g addition of rumen-protected niacin (RPN) to the diet per day resulted in increased perspiration and reduced body temperatures of lactating dairy cows exposed to HS (Zimbelman et al., 2013). In addition, thiazolidinediones (TZDs) can increase HSP production and enhance glucose utilization and energy metabolism, while dietary betaine could be a better choice in heat-stressed lactating cows (Sammad et al., 2020). Betaine plays an essential role in growth, lactation, protein synthesis, and fat metabolism in animals (Eklund et al., 2005) by acting as a methyl donor and organic osmolytes (Engin and Hotamisligil, 2010). It has been observed that supplementation of betaine in diets results in increased body weight gain and fat deposition in steers (Bock et al., 2004). Betaine supplementation in lactating dairy cows has been shown to improve milk production and composition, especially milk protein content (Peterson et al., 2012; Zhang et al., 2014). Additionally, reduction of ketone bodies such as NEFA and β-hydroxybutyrate (BHB), both markers of metabolic diseases, has been observed in lactating dairy cows fed with betaine supplementation (Wang et al., 2010; Monteiro et al., 2017). In addition, injection with growth hormones, such as recombinant bovine somatotropins (rbST), can improve productivity in heat-stressed cows (Wheelock et al., 2010) by increasing NEFA turnover (Sammad et al., 2020). Injecting rbST normally enhances the metabolic profile and boosts the immune response of stressed dairy cows (Silva et al., 2015).

It has been shown that chromium (Cr) supplementation improves energy metabolism and production in lactating cows subjected to HS (Ribeiro et al., 2020). In recent years, there has been an increasing interest in the use of Cr to reduce different stresses in high-producing livestock due to its association with glucose tolerance factors (Pechová et al., 2002). At the cellular level, Cr, in addition to apo-chromodulin, produces chromodulin, which activates insulin receptors such as tyrosine kinase and membrane phosphotyrosine phosphatase (Davis and Vincent, 1997), and presumably promotes insulin signaling (Pechová et al., 2002). Increased levels of glucose and insulin turnover due to stress could lead to elevated blood cortisol levels, as well as other glucoregulatory hormones. Cortisol, an anti-insulin, prevents glucose consumption by muscle and fat cells to preserve it for use by the brain and liver in non-ruminants, and for use by the mammary glands of lactating cows. The importance of Cr for insulin function efficiency suggests that Cr can enhance both liver metabolism and health during high metabolic demands during early lactation and peak lactation periods, along with exposure to external heat overload (McNamara and Valdez, 2005).

#### The Role of Polyphenols in Mitigating Heat Stress in Dairy Cattle

Heat stress causes oxidative stress in transition dairy cows, as revealed by various studies (Bernabucci et al., 2002). High production of free radicals and reactive species, as well as decreasing antioxidant defense, results in oxidative stress, which damages biological macromolecules and disrupts normal metabolism and physiology (Trevisan et al., 2001). Oxidative stress usually starts once reactive forms of oxygen are produced faster than they can be safely neutralized by antioxidant mechanisms (Miller et al., 1993). These conditions can cause health disorders in cattle (Miller et al., 1993).

Alleviation of oxidative stress can be achieved by supplementation with antioxidants (Sretenovic et al., 2007). Various antioxidants have specific functions as a defense mechanism against HS-induced oxidative stress (Surai et al., 2019). These antioxidants include carotenoids, polyphenolics, trace elements, and vitamins (Sun et al., 2019). Secondary plant metabolites such as polyphenols have been shown to exert anti-inflammatory and anti-oxidative effects (Gessner et al., 2017). Due to the high relevance of oxidative stress and inflammation in livestock animals, the use of polyphenols as feed additives was proposed to combat and alleviate the adverse effects of HS (Gessner et al., 2017). Supplementation with polyphenols has become popular because of its efficiency in animal nutrition (Miliauskas et al., 2004; Zhong and Zhou, 2013; Yang et al., 2015). Tannins are the most studied compounds among different types of extracts because of their free radical scavenging potential for more stable and less toxic structures by donating electrons (Maqsood and Benjakul, 2010; Huang and Xu, 2018). It is a natural antioxidant that is composed of a complex group of water-soluble polyphenolic compounds that occurs during plant metabolism. To form resonance-stabilized phenoxyl radicals, one or more aromatic rings with several hydroxyl groups can combine with free radicals. This structure has strong antioxidant properties (Rice-Evans et al., 1996). Moreover, tannin supplementation could increase antioxidant enzyme activities and inhibit lipid peroxidation in the plasma and livers of transition dairy cows (Liu et al., 2013).

A moderate but consistent downregulation of up to 25–65% in the transcription of genes involved in inflammation and endoplasmic reticulum (ER) stress in the liver was reported for dairy cattle fed with polyphenol-rich grape seed and grape marc meal extract (Gessner et al., 2017). The same study also showed that feeding this type of diet resulted in a significant downregulation of a key marker of ER stress, the fibroblast growth factor (FGF-21), which also plays a role in fat accumulation in the liver, and that supplementation with polyphenol-rich products could promote improved milk performance, probably due to the suppression of inflammation and ER stress in the liver. Another related study presented similar results wherein, even though some genes involved in inflammation and ER stress were not significantly downregulated, a certain group of cows with significant downregulation of FGF-21 presented reduced fat content in the liver and increased milk performance after feeding with a polyphenol-rich plant product (Winkler et al., 2015). Because the level of dry matter intake between cows fed with the plant product and the control group was the same, it was considered that the improved utilization of energy for milk production could potentially be due to the reduction of ER, inflammation, and metabolic stress in the liver, resulting from the supplementation of plant products rich in polyphenols (Gessner et al., 2017).

### Long-Term Strategies to Minimize Heat Stress

#### Genetic Selection

In dairy cow breeds, genetic selection for high levels of milk production has reduced heat tolerance; thus, the identification of heat-tolerant animals among high-yield breeds through recordings (Amamou et al., 2019) and phenotyping (Carabaño et al., 2019) are advantageous. During HS, dairy cows must be efficient in sustaining high production and survival rates (Gaughan et al., 2009). Most studies have focused on modeling the genetic component of performance under high heat loads to determine the genetic value of heat tolerance of animals, as described by Ravagnolo and Misztal (2000) and Carabaño et al. (2019). In this approach, the genetic component of the reaction to HS in performance is described by a broken line model (Carabaño et al., 2019). This model includes two parameters: (a) the thermoneutrality threshold and (b) the slope of decay in production after passing this threshold as a consequence of HS (Bernabucci et al., 2014). Alternatively, the use of polynomials to describe the norm of reaction of milk production across the heat load scale was proposed by Brügemann et al. (2011); Menéndez-Buxadera et al. (2012), and Carabaño et al. (2014). Compared to the broken line model, polynomial functions provide a more flexible approach and allow for a smoother transition from thermotolerance to HS, instead of an abrupt change after the thermoneutrality threshold (Carabaño et al., 2019). In addition, instead of a constant slope of decay in the broken line model (as might be expected to occur in reality), polynomial functions accommodate steeper slopes at higher temperatures (Carabaño et al., 2019). To measure heat tolerance in either dairy or meat-oriented production, reaction norm models using performance (both productive or reproductive) records and meteorological information must be extensively applied (Menéndez-Buxadera et al., 2012; Biffani et al., 2016; Bradford et al., 2016).

In addition, determination of the genetic component for other measures of heat tolerance has been mainly focused on body temperature and respiration rate (Dikmen et al., 2014; Shabat et al., 2016; Gourdine et al., 2017). The heritability estimates ranged from 0.17 to more from 0.30 in the dairy cattle study (Carabaño et al., 2019). Nonetheless, genetic variability has been detected using this type of measure for heat tolerance, although selection can be theoretically feasible but impractical because of the high cost of measuring these parameters (Carabaño et al., 2019). That’s why up to now, the efforts to produce genetic evaluations to select heat-tolerant animals have been on the basis of analyses of performance under HS (Carabaño et al., 2019). Examples of these efforts can be found in dairy (Bohmanova et al., 2007) and beef cattle (Bradford et al., 2016). A genomically enhanced evaluation has also been developed for dairy cattle in Australia (Nguyen et al., 2016).

Quantitative genetic studies, which are reinforced by a number of studies including “omic” information that focused on understanding the genetic mechanisms underlying the animal’s response to heat, are suggested to be non-negligible genetic components of thermotolerance (Carabaño et al., 2019). According to previous studies, there are three main types of focus on omics studies: (1) association studies of polymorphisms at specific genes and genome-wide association analysis (Macciotta et al., 2017); (2) genome comparison between adapted and non-adapted breeds/species to harsh environments (Chan et al., 2010) and (3) differential expression analyses (Chauhan et al., 2014b). Generally, genes reported for these three types of studies are functionally classified into similar gene ontology terms, which is a form of validating that the genome wide association analysis points the correct genomic regions (i.e., those that show differential expression under HS vs. thermoneutrality) (Carabaño et al., 2019). Moreover, the pseudo-phenotypes used to assess heat tolerance and defined in different species for association studies are good alternatives and can denote the sensitivity of animals to heat loads (Carabaño et al., 2019).

Carabaño et al. (2019) highlighted the families of genes that are present in association and differential expression studies, in addition to the functional analysis of candidate genes for the regulation of the HS response. The most represented families are HSPs and DnaJs (Carabaño et al., 2019). DnaJ proteins seem to be the fundamental partners of HSP-70 and they are significant for the translation, folding, unfolding, translocation, and degradation of proteins. Genes from interleukin, chemokine, and fibroblast growth factor families were also identified. These families mostly participate in immunological and inflammatory processes, which are one of the major animal responses when subjected to harsh environments (Bernabucci et al., 2010).

The slick hair gene deserves special attention, apart from the numerous candidate genes that have been associated with the regulation of the HS response (Carabaño et al., 2019). The slick hair gene, which is located on chromosome BTA20, is responsible for a short, sleek, and glossy hair coat, has been proposed to improve thermoregulatory ability because hair length is related to convective and conductive heat loss and the hair color is associated with solar radiation absorption (Dikmen et al., 2014; Carabaño et al., 2019). Introgression of the slick hair gene has been shown to produce animals with lower body temperatures and smaller declines in production under hot conditions, which are present in Senepol cattle and some lines of highly productive Holstein cattle (Dikmen and Hansen, 2009; Ortiz-Colón et al., 2018). These slick-positive Holstein bulls are already marketed by artificial insemination companies (Carabaño et al., 2019). Conversely, slick hair may result in the inability of animals to cope with cold temperatures, which may be important in areas that include hot and cold periods.

Breeds that originated in warm areas display adaptive advantages to HS compared with breeds that originated in temperate areas (Carabaño et al., 2019). According to previous studies, breeds from warm climates show lower respiration rates, body temperature, and sweating rates, and better reproductive performance under HS compared with breeds from temperate climates (Hansen, 2004; Berman, 2011; Gourdine et al., 2017). One of the general traits of locally adapted breeds is their low level of production (Carabaño et al., 2019). It has been concluded that low productivity of locally adapted breeds might be a constitutional characteristic of these breeds since several studies show that breeds from warm climates and their crosses with selected breeds, when improved feeding is provided, tend to favor fat deposition and body condition score over milk production (Berman, 2011). Thus, improving productivity in breeds adapted to harsh conditions might be impaired by this characteristic, and, on the other hand, the use of these breeds to improve heat tolerance of selected breeds might confer an undesirable genetic background in addition to the desired heat tolerance (Carabaño et al., 2019).

#### Environmental Modification

During hot weather, it is very important for cows to undergo regular HS assessments and access adequate cooling facilities (Sammad et al., 2020). Modification of the environment could decrease heat gain and improve heat dissipation to protect dairy cattle from HS (Wang et al., 2020). Provision of houses or shade, evaporative cooling with water in the form of fog, mist, or sprinkling with natural or forced air movement, and cooling ponds are the most predominant measures to alleviate HS in dairy cows (Atrian and Shahryar, 2012). Although shade reduces heat accumulation from solar radiation, it does not have a direct influence on air temperature or relative humidity; thus, additional cooling is essential for lactating dairy cows in a hot and humid environment (West, 2003). The provision of water sprinkling with ventilation could improve evaporation, which serves as the dominant mode of heat dissipation for animals under HS conditions (West, 2003). Dairy cattle exposed to HS at approximately 36°C environmental temperature, when sprayed with water, have been reported to have a lower rectal temperature and respiratory rate than non-cooled cattle (Marai et al., 1995). On the other hand, evaporative cooling systems are used to evaporate moisture and cool the air surroundings of the cow by using high pressure, fine mist, and large volumes of air (West, 2003). Evaporative cooling is performed either by passing air over a water surface, passing air through a wetted pad, or by atomizing or misting water into the air stream. Thus, using this system will provide a favorable environment for lactating dairy cows in warm climates through the reduction of air temperature resulting from the removal of heat energy required to evaporate water (West, 2003).

Modification of the environment (such as air movement, wetting the cow, evaporation to cool the air, or shade) in lactating dairy cattle under HS will improve production efficiency, such as increased milk yield and improved fat-corrected milk yield (probably due to lower energy expenditures for body cooling), reduced respiratory rate, and decreased rectal temperature (West, 2003). These environmental modifications are based on combinations of the principles of convection, conduction, radiation, and evaporation to alleviate HS.

## Conclusion

Improving management practices to cope with increasing environmental temperatures is an important factor in the dairy industry. If the ability of the animals to cope up in such conditions is overwhelmed by extreme temperatures, adjustment in the formulation of the ration is critical to maintain the performance of dairy cattle despite reduced feed intake. HS adversely affects the function of the rumen microbiome and metabolism resulting in high lactate while decreasing the acetate producing bacteria, thus affecting the milk production. It also decreases fibrolytic bacteria that are sensitive to low pH conditions, while increasing amylolytic bacteria that are resistant to low pH conditions. HS has both direct and indirect impacts on the health and welfare of animals by impairing the rumen and intestinal mechanisms, thereby reducing the efficiency of food utilization. Regulating rumen microbial fermentation is essential for the use of nutrients and additives to alleviate the negative effects of HS. The basic physiological mechanisms of the rumen are altered by high environmental temperatures, which negatively affect ruminants, resulting in health problems and an elevated risk of metabolic disorders. Therefore, strategies are essential to mitigate HS, including short- and long-term alleviation strategies. Through nutritional management, gene selection, and modification of environmental conditions, HS can be minimized.

## Author Contributions

All authors listed have made a substantial, direct, and intellectual contribution to the work, and approved it for publication.

## Conflict of Interest

The authors declare that the research was conducted in the absence of any commercial or financial relationships that could be construed as a potential conflict of interest.

## Publisher’s Note

All claims expressed in this article are solely those of the authors and do not necessarily represent those of their affiliated organizations, or those of the publisher, the editors and the reviewers. Any product that may be evaluated in this article, or claim that may be made by its manufacturer, is not guaranteed or endorsed by the publisher.
